# Integrated Approach to Achieve a Sustainable Organic Waste Management System in Saudi Arabia

**DOI:** 10.3390/foods11091214

**Published:** 2022-04-22

**Authors:** Nibras Abdullah, Ola A. Al-wesabi, Badiea Abdulkarem Mohammed, Zeyad Ghaleb Al-Mekhlafi, Meshari Alazmi, Mohammad Alsaffar, Mohammed Anbar, Putra Sumari

**Affiliations:** 1 Department of Computer Engineering, College of Computer Science and Engineering, University of Ha’il, Ha’il 81481, Saudi Arabia; neprasf@gmail.com (N.A.); b.alshaibani@uoh.edu.sa (B.A.M.); 2 National Advanced IPv6 Centre, Universiti Sains Malaysia (USM), Penang 11800, Malaysia; anbar@usm.my; 3 Faculty of Computer Science and Engineering, Hodeidah University, Hodeidah P.O. Box 3114, Yemen; 4 School of Computer Sciences, Universiti Sains Malaysia (USM), Penang 11800, Malaysia; putras@usm.my; 5 Department of Information and Computer Science, College of Computer Science and Engineering, University of Ha’il, Ha’il 81481, Saudi Arabia; ziadgh2003@hotmail.com (Z.G.A.-M.); ms.alazmi@uoh.edu.sa (M.A.); m.alsaffar@uoh.edu.sa (M.A.)

**Keywords:** genetic-based waste collection, IoT, truck waste collection management, Zero Waste

## Abstract

Organic waste management (OWM) has always been a fundamental aspect of human populations. Approaches to OWM must be matched to the characteristics of a certain population. In this consideration, the Kingdom of Saudi Arabia (KSA) is no exception. Organizations are being aligned to focus on sustainability matters sharing significant features with universal trends, especially the integration of 3Rs (reducing waste, reusing, and recycling resources). However, the degree and nature of advancement in the direction of sustainability vary depending on the economic level of a state. High-income economies can afford to pay a higher price to integrate 3Rs technologies. Most recent endeavors have focused on achieving ‘Zero Waste’, which is costly for low-income developing countries. The expectations of OWM systems in KSA must be estimated. In this work, the situations in KSA and other countries are analyzed, and pertinent aspects are explored. Matters relating to the sustainability of OWM are conceptually assessed. This study proposes an integrated method for an organic waste management system to achieve sustainable OWM in the context of state policy and appropriate frameworks, suitable technology, institutional order, operational and monetary administration, and people consciousness and involvement. A genetic-based waste collection transportation algorithm that enhances the efficiency of waste collection truck management is presented in line with this technology. The selected routes based on the *R_fs_* and *IPv* are the most efficient among those available for the examined smart bin destinations. The minimum *R_fs_* of selected routes is less than the maximum *R_fs_* of available routes by 2.63%. Also, the minimum *IPv* of selected routes is less than the maximum *IPv* of available routes by 27.08%. The proposed integrated approach, including the waste collection transportation algorithm, would be beneficial across a variety of country-specific layouts.

## 1. Introduction

Millions of Muslims visit the Kingdom of Saudi Arabia (KSA) every year to do Pilgrimage (Hajj) and Umrah. Muslims visit holy areas of worship, such as the holy mosques al Haremeyn (Makkah and Medina) and Al-Masha’ir (Arafat, Mina and Muzdalifah) [1]. The number of pilgrims arriving in KSA for Hajj (the 12th month of the Islamic lunar calendar) and Umrah has significantly increased in recent decades due to the ongoing expansion of the holy mosques, enhanced transportation services and safety, and decreased aggregate cost and time, with an annual increase of 1.15% from 1993 to 2014 [1,2,3]. The KSA produces approximately fifteen million tons of municipal solid waste (MSW) per year, with a daily average of 1.4 kg per person. The average rate of waste generation is projected to increase to 30 million tons by 2033 due to continued population growth and civic expansion. Medina city generates approximately 887 thousand tons of MSW on an annual basis. This rate will increase due to the growing number of pilgrims every year. The highest rate of waste generation occurs during the last 10 days of Ramadan (the 9th month of the Islamic lunar calendar) due to large numbers of local and foreign pilgrims in Makkah and Medina cities [1]. The new Vision 2030 strategy in KSA aims to minimize all types of waste and to generate renewable energy from natural origins, including generated waste. The strategy has produced a roadmap for the implementation of a solid waste management system to enhance the economic and environmental importance of waste beyond recycling and reuse [4].

The increase in food waste is placing robust strain on the environment and increasing worldwide the economic value of proper management. Among the conventional technology for processing organic waste (incineration, waste processing, anaerobic digestion, etc.), composting is an economically feasible and dependable PT, regardless of technical shortcomings and social problems, and recycling technology.

Recent research describes diverse techniques to enhance PB composting, as well as co-composting, the addition of organic/inorganic collectives, the reduction of fuel line emission, and microbiological diversities.PT composting carried out using current technology quickens the deterioration of natural waste, produces value introduced mature compost, saves costs, and is technically possible for PT composting [5].

Many researchers, such as Nizami et al., Rehan et al., Miandad et al., Khan et al. and Ouda et al. have presented various solutions to the waste elimination problems of KSA, in line with the country’s vision for 2030. These solutions have included producing energy and value-added outputs, such as chemicals, organic fertiliser and nutrition, from different domestic waste origins through waste recycling [1,6,7,8,9]. In recent years, waste recycling has gained widespread recognition for its ability to keep products and materials in use, which can then be utilized in extraction, natural systems regeneration, transportation, and production of new raw substances, thereby enabling the circular economy in countries like KSA [1].

Composting natural waste merchandise on agricultural land is considered one of the maximum economical, practical, and environmentally useful control options. As a rule, the system includes the herbal organic decomposition of natural waste additives and various species of microorganisms [10]. The generation of food as a type of organic waste is a natural result of human behavior. The elimination of such waste is necessary to improve the quality of life. Organic waste management (OWM) mechanisms were firstly designed to remove waste from close proximity to living areas to maintain public health. After understanding the risks of uncontrolled disposal, procedures to remove waste were designed and fundamentally implemented. Diverse substances and energy recovery mechanisms have been designed and recently included in modernistic systems. Waste management systems must now be redirected towards sustainability in international undertakings.

Furthermore, adhering to the United Nations’ Sustainable Development Goals to minimize malnutrition and extirpate starvation necessitates effective management and proactive efforts to ensure sustainable food security. The Group of Twenty (G20) is an intergovernmental forum of large and advanced economies (19 countries and the European Union) that plays a pivotal role. G20 members comprise 60% of citizens, 80% of economic production and 75% of greenhouse gas (GHG) emissions [11]. This commercial success has a negative effect on the environment. While the G20 nations are preparing for the coming decades, this is an opportunity to use their experience to set an example for other nations around the world.

**Figure 1:**
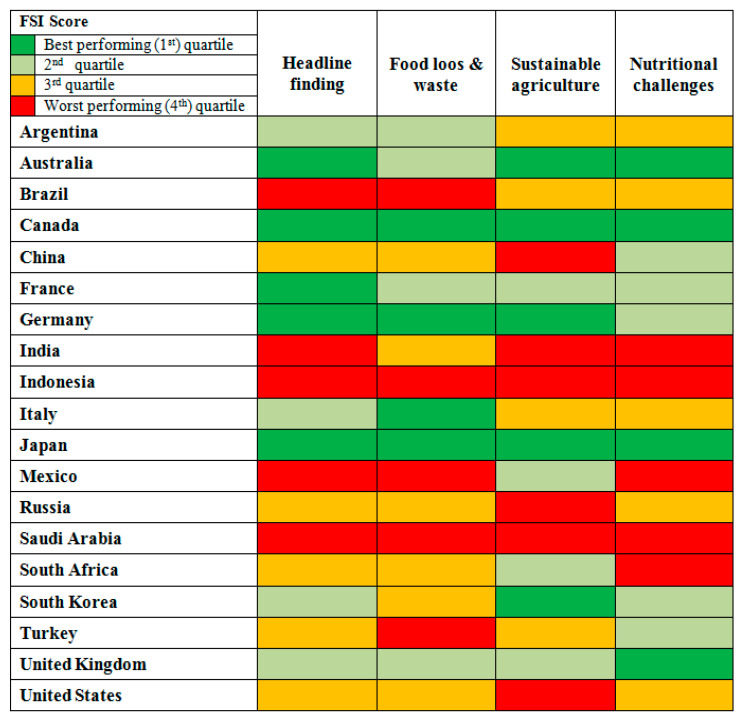
FSI results—G20 countries.

Japan, Germany, Canada, France and Australia are amongst the G20 members who performed best in the Food Sustainability Index (FSI) in 2021. Although these nations still have room for improvement, they combine great production with a strong strategy. Techniques for dealing with food scarcity and waste are well understood amongst the group. However, more procedures can be carried out to execute the mandated law that holds all stakeholder economies accountable. Figure 1 presents the FSI score results of the G20 countries, according to the Economist Intelligence Unit and the FSI 2021.

The significance of this study is in finding solutions to reduce pollution and maximize resource use, especially organic waste management systems, and utilizing this technology to achieve the highest levels of waste management. The research’s significance extends to the development of a methodology for implementing these solutions. This research proposes an integrated organic waste management approach for KSA, to enhance waste management performance.

Related studies in waste management mainly focus on solid waste management, which encompasses different waste types in addition to food waste. Some researchers have used IoT architecture to enhance waste management performance. However, this study addresses a variety of issues.

The issues are as follows:

- The sources of organic waste are not specified and clearly classified.
- The management of organic waste using IoT is not properly addressed.
- Transport lacks the use of intelligent algorithms.
- Public awareness of the law.

This research aims to address the above-mentioned pressing issues of source waste generation, collection and transportation in Saudi Arabia, by using the proposed integrated organic waste management system.

## 2. Food Waste Is a Problem

According to the World Bank report in 2020, more than two billion tons of MSW were produced annually, with at least 33% of this being ecologically harmful. On a global scale, the average amount of waste produced per individual each day is 0.74 kg. These values range from 0.11 kg to 4.54 kg. Despite accounting for just 16% of the world’s population, high-income exports account for approximately 34% or 683 million tons of global waste [12,13].

Waste generation has enormously increased across the world in the last decades, and there is no indication of it decelerating down. Global MSW generation is expected to have increased by 70% to 3.4 billion metric tons by 2050. This situation is a result of a number of factors, such as world population growth, urban expansion, economic evolution and consumer shopping behavior. People generate millions of tons of waste each year, and this is rapidly becoming a worldwide problem. The necessity for authorities to provide appropriate waste remediation and disposal facilities has become ever more significant with the massive quantities of waste accumulating. However, less than 20% of waste is recycled each year, with massive aggregates still transmitted to landfills. Waste is frequently disposed of in hazardous open tips, particularly in developing countries. High-income countries produce more waste than low-income poorer countries, but they often have better waste management systems to help address these issues [14].

**Figure 2:**
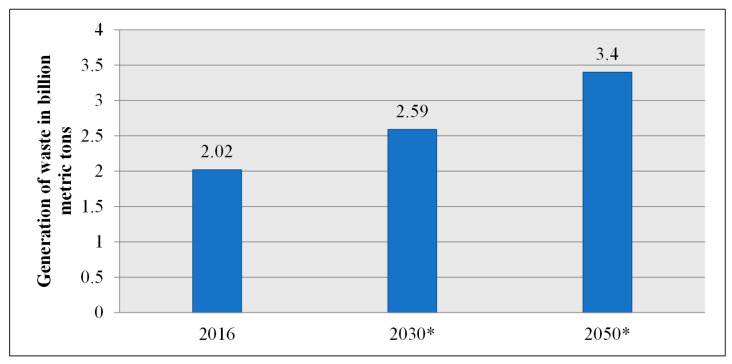
Worldwide municipal solid waste generation planning (2016–2050) [15]. *: Prediction values.

Waste generation statistics by billions of metric tons are presented in Figure 2. These statistics clarify the amount of MSW produced globally in 2016, with projections for 2030 and 2050. According to estimates, approximately 3.4 billion metric tons of MSW will be produced globally.

The East Asian region, the Middle East, North Africa and the Pacific region produce the greatest volume of waste, but produce the least in absolute terms. The whole waste area of the fast-developing areas of Sub Saharan Africa, the Middle East, North Africa and South Asia are predicted to more than triple, double, and double by 2050, respectively. More than 50% of waste in these areas is currently overtly thrown, and the trend of waste accumulation will have huge ramifications for the environment, health and economic growth, necessitating immediate action [2,3].

Organic waste accounts for more than 50% of global MSW, and is a serious problem in many regions. The East Asia–Pacific (EAP) region produces the most organic waste, accounting for approximately 62%, followed by the Middle East and North Africa (61%) [12].

Organic waste recycling is one of the strategies for solving this problem. Moreover, different methods are available for utilizing organic wastes, such as composting for fertilizer production, and anaerobic digestion (AD) for energy production [16,17].

Vlachokostas et al. proposed a scheme that included a Region for Biodegradable Waste Treatment Installation. The distance to the municipality is two km, and it is seven km to the slaughterhouse [18]. This public place is effortlessly approachable and also is even in near proximity to a municipal vegetable garden. The chosen place was agriculturally intense, with focused production of biodegradable waste. The study examined different types of organic wastes, including manures, hygienized slaughterhouse waste, cheese whey, rotten potato pulp, and olive mill residues. These could be gathered from their disposal places and transported using boxes and heavy obligation vans to the Biodegradable Waste Treatment Installation center. Hygienization is a crucial factor in the remedy of such waste, specifically stomachs, animal fat, and blood [18].

Moreover, modern linear agricultural manufacturing structures make it feasible to supply meals in large portions for a developing population. However, this leaves a quantity of agricultural waste to be discarded or recycled for reintroduction into the manufacturing chain for new uses. To obtain this goal, numerous processes for handling meals waste were explored [19].

The gathering and transportation of waste are primarily based on predetermined paths, ensuing in needless costs and wasted equipment. Empty packing containers are often gathered first, even as waste-stuffed packing containers spill onto the road. This spillage might additionally bring about elevated cleaning costs, health and safety dangers, and court cases from residents.

**Figure 3:**
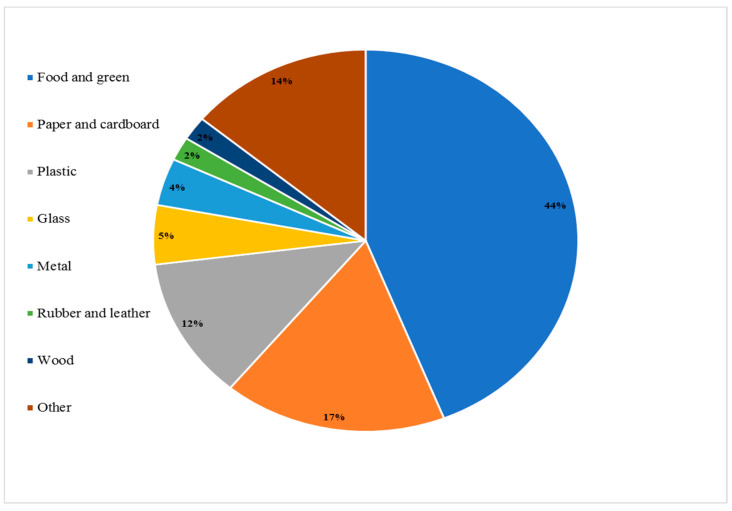
Classification of municipal solid waste produced globally in 2016 by the substance class [12].

Figure 3 presents the classification of MSW produced globally according to substance class, with food and green waste accounting for 44%.

According to UNEP, statistics are distributed in an inequitable manner amongst nation income classes and regions. Based on a regional level, developed economies in Australasia, North America and Northern and Western Europe have made the most progress in data collection in the sectors of household, food service, and retail. Meanwhile, numerous developing economies in the Caribbean, Latin America and Africa remain data-deficient [20].

However, the UNEP data challenged the conventional view that food waste is a problem that only affects advanced and high-income nations, and instead emphasized it as a global concern. Accordingly, the most important obligation in minimizing food shortage and waste is to recognize the problem. Hence, people should be more aware of the scope of the problem, the amount of food wasted, and the manner food is utilized. The first step, and a form of protection, is to estimate the costs [20].

**Figure 4:**
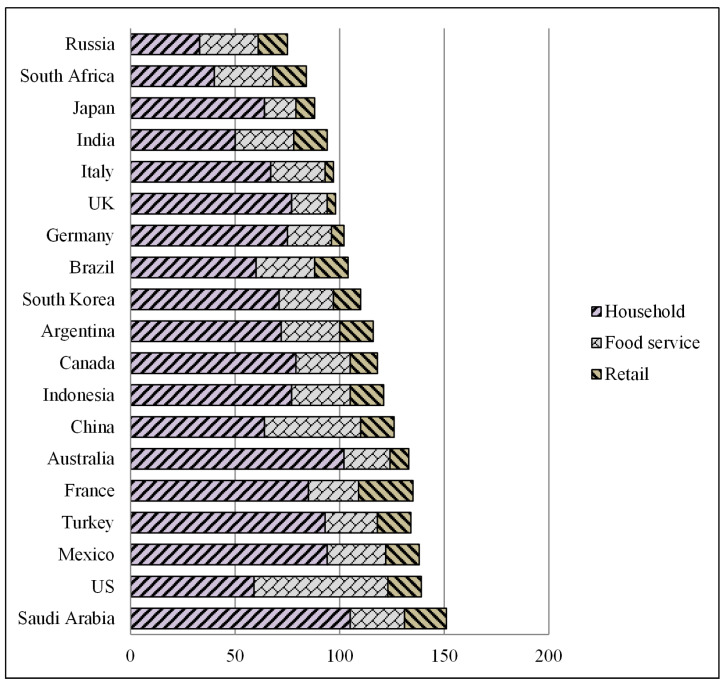
Food waste index flowchart (kg/head/year) 2021 [20].

Although UNEP admits that their data are weak in terms of numbers, information is more abundant in advanced economies, implying that it still provides relevant information for the FSI. Household waste is frequently greater than the aggregate of other food waste categories (retail and food service waste). The US is an exception due to the preferences of the American people and a and culture of dining outside the house. The G20 nations that perform well in all three categories are Japan, which ranks in the top six for all three categories, Italy, the UK and Germany, in spite of the latter two each having a task to work on reducing household waste, as shown in Figure 4. However, Saudi Arabia and France are particularly short on household and retail waste. Mexico and Turkey also rank poorly, as presented in Figure 4.

According to the retail waste index, France came in last place, noteworthy because the French government has been paying greater attention to this issue since 2016. Legislation was passed that required retailers with a certain inventory size to partner with a company to distribute unsold food for free or face a penalty. However, France’s legislation may have resulted in a more careful assessment of food waste from retail establishments. A possible situation is that food waste is under-reported. Legislation like this may be a more appropriate model that some other nations lack. Japan imposed penalties for food organizations that violated recycling and reuse legislations in 2020. Such laws are not imposed in the UK.

Certain religious and cultural elements can play a critical role in reducing food waste. In many cultures, serving a large amount of food during hospitality is essential to be a gracious host. Accordingly, changing this mindset and consciousness to minimize food waste can be difficult. However, governments should raise awareness and take steps to align with current trends to ensure that the lawful system is a viable choice [21].

As previously mentioned, the oldest and largest religious gatherings in the world happen every year in KSA. Millions of Muslims from all over the world do Pilgrimage to worship at Al-Haram (Holy Mosques in Makkah and Medina) and Al-Masha’ir (Mina, Arafat and Muzdalifah) [1]. The Al-Haram mosque in Makkah has a total size of approximately 356,800 km^2^, which includes indoor and exterior prayer areas. More than two million Muslims gather to worship during Ramadan and Hajj [22]. Garbage collection and disposal are major tasks for the authorities during the Ramadan and Hajj seasons, which result in a significant increase in waste production in a short amount of time. Moreover, this situation becomes more challenging due to the combination of rising food waste and plastic waste [23]. Most waste is disposed of in landfills that lack leachate and landfill gas collecting methods [24,25]. This activity results in water and soil pollution and emission of huge quantities of GHGs [9]. Hence, the demand for an appropriate waste management system in Makkah city has intensified to deal with the enormous quantities of waste produced by the local population and the pilgrims [1].

In KSA, garbage is collected using private or social funds and disposed of in landfills. The waste management system in Saudi Arabia lacks waste disposal services and transfer charges. In the next 10 years, most landfills are expected to be full and unable to be used. Although recycling, energy recovery and reuse are often considered, they are still at an early phase. Waste recycling and sorting are powered by an effective unofficial sector. The recycling average ranges from 10% to 15%, mainly due to the existence of the unofficial sector that reuses paper, plastics and minerals from domestic waste [26].

The existing collection system in populated urban zones increases the expense of installing air systems in residential areas. Moreover, restricted placement of trucks and waste bins causes issues in the operation of the collection system, especially in historical areas.

The collection of organic waste is based on pre-determined paths, resulting in unnecessary expenses and a lack of equipment. Empty containers are frequently collected first, whilst waste-filled containers leak onto the street or onto a lot, which may result in increased cleaning costs, health risks and complaints from residents.

This research focuses on IoT technologies in the OWMS and proposes an effective waste management based system based on the IoT to improve waste collection activities in the waste source area, an important stage, especially for recycling waste in KSA’s urban areas.

The increased functionality of cloud services, applications and databases will make communication easier amongst different IoT gadgets, forcing new communications between the existing system and the new system. The ensuing data networks will minimize expenses and dangers and enhance waste management operations. The IoT architecture is predicted to reduce waste collection operating expenses in Saudi Arabia, through the use of collected data shared between bins, smart containers and vehicles, to allow automation and coordination of the identification of waste for recycling and waste processing. The implementation of future Internet technology enhanced by the utilization of the Internet Protocol on many wireless sensors permits the IoT paradigm.

Many sensor units can be considered to be a component of Wireless Sensor Networks (WSNs) when utilized in a city. These sensors collect and process ambient information to improve legacy city infrastructure, which is referred to as smart cities [27].

## 3. IoT-Based Waste Management Systems

A variety of waste management models have been produced using specific IoT devices. This part of the study presents the most common IoT-based waste management techniques.

Various models employ IoT gadgets, including capacity, weight, temperature, humidity, and chemical sensors, depending on the need for these devices.

Mustapha et al. presented a MSW stage using recycling collection data based on IoT Innovation. The study involved a demonstration of waste collection, transportation, reuse and processing [28]. Chaudhari et al. presented an option for strong waste recycling, which involved reusing materials and energetic optimization. An choice of factors was created and thereafter the quality of waste in each container was compared on a daily basis [29].

Chaudhari et al. presented a monitoring system to monitor bins and trucks via RFID and ICT, utilizing cameras and GPS. The bin used for variant waste types is located outside the doors. Homogenous and heterogeneous trucks are used for waste collection [28,29]. The absence of a DSS for determining choices in actual time, IoT equipment, such as RFIDs and actuators, or the association of a single sort of sensor are among the fundamental aspects [29]. Gupta et al. recorded the collection of bins, for studying and enhancing waste collection. The article explains the design and implementation of the waste disposal system needed to demonstrate the significance and diversity of the facility’s value [30]. The system assigns zones to increase the efficiency of nutrients, and reflect people’s lifestyles at different times of the year. The system has a smart structure and framework for the use of data in statistical communication processes.

Anagnostopoulos et al. have proposed a system in which WSN is used to inform the driver training process. The study displays the WSN model as a technology that allows for the active implementation waste collection in towns. This system is designed to improve performance and supervise waste transportation to the site through the collection process [31].

Vitorino de Souza Melaré, et al. presented a collection-monitoring model for early detection and evaluation of waste by sensory bins. Their research described a new application using distributed sensor technology and GIS to observe and track MSW. They also presented an energy use model for improving solid waste collection that can be used in big cities [32]. It provides three models for the optimization of dynamic scheduling routing. Di Maria and Micale proposed a model for analyzing the effect of intensity of solid waste source isolation on collection costs and fuel consumption [33].

Mak et al. examined the economic performance of pneumatic and door-to-door waste collection systems in real city settings, presenting hypothetical analysis of how the pneumatic waste collection system compares with the door-to-door truck collection system. Intensive pneumatic systems and door-to-door collection have different disadvantages in urban areas. The cost of using pneumatic systems in existing residential zones is increased by urban infrastructure and buildings [34].

Adeyemo et al. offered an automated waste collection system according to a Ubiquitous Sensor Network, presenting a new model for collecting MSW in residential and business buildings using IoT technologies. The capacity sensors are the most IoT-enabled technologies, followed by RFIDs and weight sensors [35].

Agricultural food waste is generally available and inexpensive. In addition, its intake is predicted to have remarkable long-term effects on network and financial improvements, through means of reducing environmental pollution, reducing immobile lipase charges, and in the long run achieving downside potential. Expenditure on final goods is reduced in a healthy and safe manner through the process of bio catalysis. Also, recycling the waste of agro-food enterprises is one of the foundations of the sustainable financial system [36]. According to the above review, the majority of proposed models failed to consider the kind of waste and the effect on public health of different types of waste. Moreover, the authors believed that waste cannot be re-used. Although not all waste kinds can be re-used, some types of waste can be utilized to their full potential as raw material. Organic waste can be used for renewable energy systems.

Private waste or household rubbish, such as leftover foods, vegetables peels and natural products, polythene and paper, can be reused. Organic waste can be used for renewable energy systems. Furthermore, surplus peels and vegetables can be utilized as fertilizers, whilst polythene can be sold to major purchasers in advertisements. This situation is only possible if we take responsibility for separating our waste. Separation at home is also simple for housekeepers and laborers. Employees who handle waste work in severe and exhausting conditions to physically segregate the waste, which may result in health issues. Thus, the waste must be isolated at the source.

Waste types can be specified based on their source. For instance, organic waste can be located in markets, restaurants, houses, agricultural areas, etc., whist solid waste can be located in construction places [5]. Moreover, smart algorithms for routing and gathering are not used in the transportation of waste [37,38].

Smart cities are densely inhabited and urbanized, so waste is difficult to collect using standard trucks, especially during peak hours. However, the type of waste determines the size of the trucks. Thus, small trucks will reduce traffic and make it easier to move around the city.

## 4. Materials and Methods

This research proposes the construction of a waste management system integrated with four main factors as follows: Education: Educated Community; Community: Public Participation; Government: Law and Policy; and Appropriate Technology.

**Figure 5:**
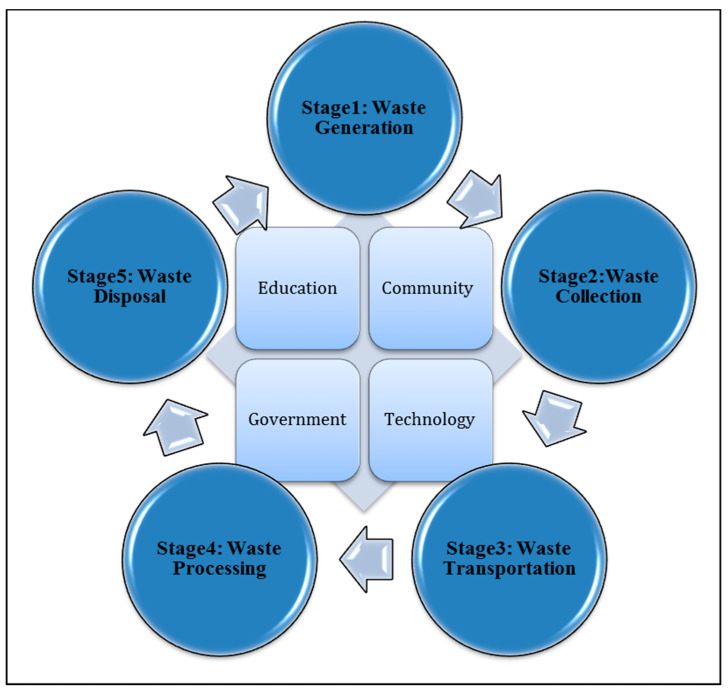
Integrated approach for organic waste management.

The proposed integrated system includes waste generation to resolve and treat numerous waste issues by identifying organic waste resources. The waste collection uses IoT technology by providing smart bins that are utilized to enhance waste collection and supply information for statistical analysis. Waste collection smart trucks are utilized to enhance the transportation of collected waste. Waste processing and waste administration centers are used for data analysis. The final waste disposal stage is decision-making. Figure 5 shows the integrated approach for organic waste management.

### 4.1. The Architecture of the Waste Management System

**Figure 6:**
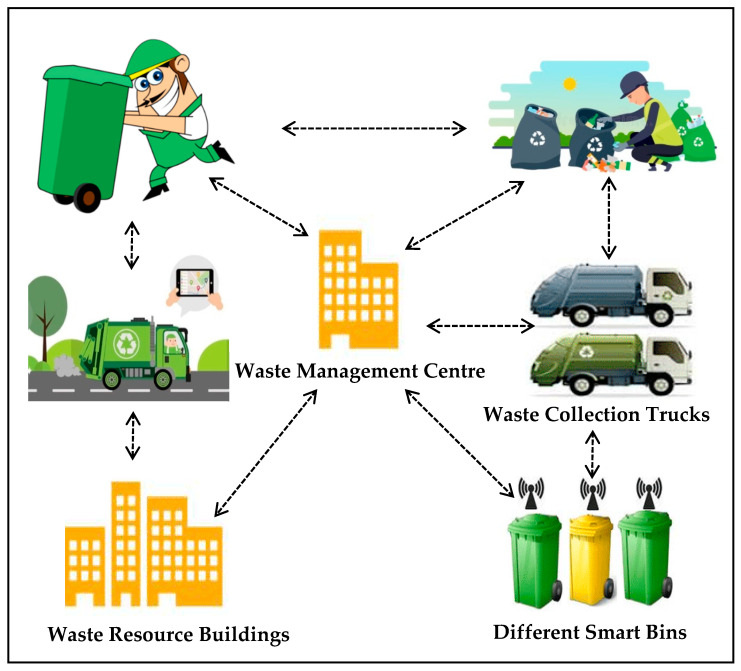
Waste management system architecture.

The architecture of the waste management system is shown in Figure 6, which includes waste resource building, a waste management center (MC) for handling diverse waste issues, smart bins with IoT technology to enhance waste collection and provide information for collection decision-making, and waste collection trucks.

#### 4.1.1. Waste Generation

Waste source locations, such as homes, shops, restaurants and supermarkets, have unique IDs and priority averages, depending on the situation of the waste and the significance of the site, such as hospitals and schools. Priority is based on different factors, including place category (medical center, school, accommodation, or market), type of waste (e.g., mixed food, vegetables, meat, and medication) and the level of danger of the waste type. Organic waste resources are categorized as follows:

- **Residential areas:** Blocks, compounds and hostels;
- **Commercial establishments:** Hotels, supermarkets and restaurants;
- **Medical organizations:** hospitals and medical centers;
- **Educational buildings:** Schools and universities;

As shown previously in Figure 4, households in KSA produce more waste food compared with food services and retail than in other countries, indicating that we should pay more attention to the household and educate residents about the importance of waste management systems.

#### 4.1.2. Waste Collection

At this stage, smart bins send the information about the waste to the waste MC via the cloud system by using GSM or GPRS according to the area infrastructure.

The bin will call the waste MC to send a truck to collect the waste only if the bin attains its threshold weight, or if the bin is full. The bin is considered full when its level exceeds or is equal to 90%.

**Figure 7:**
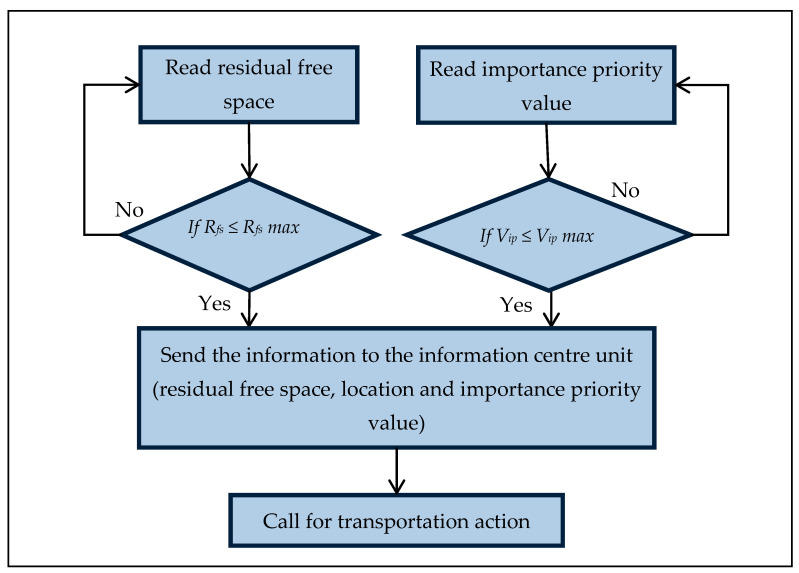
Intelligent bin handling information.

The system dispatches the information from the bin to the waste collection transport. The system then supplies a weighted priority in status, which overrides the threshold value. Subsequently, the system locates the most suitable truck with an optimal route to gather the waste by utilizing a smart routing algorithm. Figure 7 presents the intelligent bin handling information according to the following equations:

If *R_fs_ ≤ R_fs_ max* → then send the information to perform an action; else, read the residual free space

or

if *V_ip_ ≤ V_ip_ max* → then send the information to perform an action; else, read weight;

where *R_fs_* is the residual free space, *R_fs_ max* is the threshold residual free space, *V_ip_* is the weight measure, and *V_ip_ max* is the threshold importance priority value.

#### 4.1.3. Waste Transportation

This work proposes a transport algorithm as part of an integrated approach for organic waste management. Collecting waste utilizing normal trucks through peak hours is difficult during peak hours because smart cities are densely inhabited and urbanized. The volume of collection trucks is determined based on the type of waste. Small trucks are proposed for waste collection because they can help in minimizing traffic and can more simply move around an urban area. Trucks are utilized to collect wastes from their source and transport them to a disposal area where they can be used for renewable energy systems, to a recycling center, or to a landfill site. Every truck is linked to a GPS/GPRS. This technique suggests various truck sizes depending on the zone and expected waste-producing volume. Thus, the location of the truck and bins are determined.

**Figure 8:**
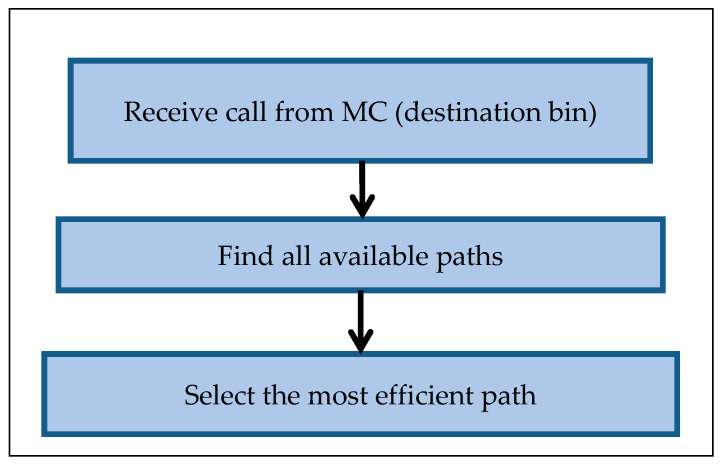
Process of the transportation system.

The transportation system proposes an efficient algorithm for collecting organic waste from the specific destination bins. In the same time, it will collect other bins with a status close to full. Figure 8 presents the transportation system process as follows:

The destination bin ID and location are received from the waste MC. The system calculates all the available paths from the truck origin location to the destination bin location by using a genetic algorithm (GA) based on the transportation network and bin distributions. Then, the efficient path is selected according to the full status for each bin amongst every available path. The collection decision-making for each bin is calculated by the MC according to the available information received from the bins.

A proposed transport algorithm for collecting full waste bins is based on the residual bin space for waste as the first constraint parameter, and the consuming time of collecting waste for the same waste bin as the second constraint parameter.

The transportation network map information is formulated as follows:

Rout (edge) *e*(*u*, *v*): *u* ϵ *V*, *v* ϵ *V*, and all link *e*(*u*, *v*) ϵ *E*.

The constraints parameters are as follows:

Residual free space (bin_id) = *R_fs_*(bin_id) < *R_fs_ max*: *R_fs_ max* is the maximum residual free space in the bin that is considered to be ready to collect.

Importance priority value (bin_id) = *V_ip_*(bin_id) < *V_ip_ max*: *V_ip_ max* is the maximum importance priority. Low value is important to clean the bin.

The route from the truck position (S) to the target bin (T) is defined as a sequence of other bins that are considered ready to collect.

*R_st_* (S,T) = bin_1, bin_2, bin_3, …, bin_*t*, when all bins are on the same route.

*RR_fs_* (S,T) is the route residual free space for all bins from the truck position to the target bin.

*RV_ip_* (S,T) is the route importance priority value for all bins from the truck position to the target bin.

(1) $$Rfs{\left(R\left(vs,vt\right)\right)}^{}=\sum_{i=s}^{i=t}Rfs(\mathrm{bin}\_i):\;\mathrm{bin}\_i\;\epsilon \;R_{st}\;\mathrm{and}\;1\;\leq \;i\;<\;n$$

The cost for each path is computed as follows:

(2) $$RVip{\left(R\left(vs,vt\right)\right)}^{}=\sum_{i=s}^{i=t}Vip(\mathrm{bin}\_i):\;\mathrm{bin}\_i\;\epsilon \;R_{st}\;\mathrm{and}\;1\;\leq \;i\;<\;n$$

On the basis of Equations (1) and (2), we calculate the fitness function as follows:

(3) F(R) = Max(RVip(R(vs,vt)), Rfs(R(vs,vt)))

The waste MC provides this information based on data received from smart bins around the city. The proposed algorithm in this experiment responds to the call from the specific target bin that needs a waste collection truck. The algorithm considers all the bins that have less residual free space on the way to the target bin, by using GA. According to the available information on the road map, the sequence procedural steps of the GA are as follows:

Step1—The population is randomly assigned to one of the available transportation paths, based on the bin’s residual statuses. Each path has information on how to deal with the case.

Step2—Assessments: The available transportation paths in a population are assessed by utilising the fitness function to locate the most efficient route that includes most full waste bins needed to collect.

Step3—Selection: Individuals are chosen based on the classified fitness value.

Step4—GA process: The route is converted to enhance the solution.

Step5—The process is terminated if the transportation path includes all the bins that are ready to collect on the way to the target bin.

Step6—The population is changed: After route alteration, the worst route is avoided and changed with a new route.

Steps 2, 3, 4, 5, and 6 are iterated until termination.

In the experimental study, the area size was assumed to be 1000 m^2^, and the smart bins were randomly distributed. Each bin had a location, status (full/not-full/can collect) and the expected time it takes to collect the waste from this bin. The experimental evaluation is presented in the experimental results section.

#### 4.1.4. Waste Processing

**Figure 9:**
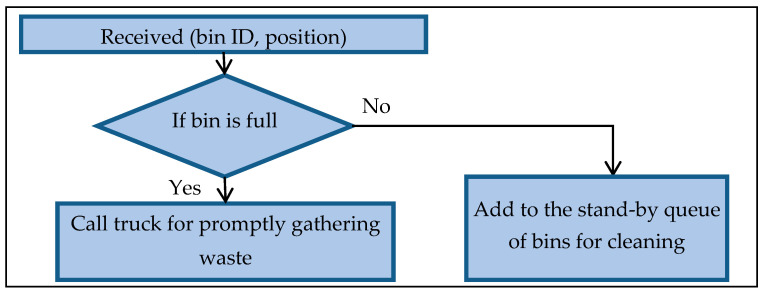
Proceedings for the decision-making process.

The waste management station sends commands to the smart trucks according to truck location and the information available from the smart bins. Figure 9 presents the proceedings of calling trucks for waste gathering.

The database of smart bins is hosted on a cloud computing infrastructure to supply high data availability. The composite equipment in the bins allows real-time data collection. The waste management station analyses the assembled data from the IoT equipment and provides helpful information for decision-making inside the intelligent waste management system.

#### 4.1.5. Waste Disposal

Waste disposal differs from natural methods because the organic waste is a source of renewable energy.

The proposed architecture of the waste management system is integrated with four factors: education, community, government and technology.

### 4.2. Education

Consciousness-raising and information distribution are offered to enhance recycling presentation, and are crucial for recycling platform success [39,40]. The researchers conducted seminars and brainstorming meetings for the sessions to provide information on waste administration platforms, such as the advantages of waste sorting, sorting procedures and how to gather the sorted waste, especially for separated organic waste. The meetings also concentrated on the issues and hurdles, giving advice on waste sorting to optimize its benefits.

It is necessary to pay more attention to operating educational campaigns to notify the public about the quantity of wasted food in the nation in household, food services and retail contexts. These campaigns should inform the public about the choices available to them to participate in improving the situation (e.g., how to obtain domestic food donation NGOs, how to avoid excess food offering and how to minimize wasted food at home). Moreover, these campaigns should provide solutions to guarantee that food waste goals and planning are incorporated into outlines of responsibility, and should state methods of food waste conversion. An educated community is more aware. Institutions should focus on enhancing the effectiveness of the food management system, in which private sector involvement should be incorporated when possible. Therefore, the suitable institutions must be organized by firstly determining the functions of each organization inside the system.

### 4.3. Community

The committee must conduct studies to measure people’s willingness to help in reducing wasted food and choose regions with the least attention or compliance. Gathering a sympathetic audience to the cause to encourage good behavior is also necessary. Furthermore, popular knowledge campaigns and supporting government activities must be launched to raise awareness about the extent of the problem of wasted and lost food, and to authorize and guide urbanized society to take the required actions. Moreover, the community should identify charities that are working in areas of wasted and lost food and consider whether they can be provided with public and private funding to expand their actions.

### 4.4. Government: Law and Policy

Each nation should have an incorporated sustainable organic waste administration, that involves aspects such as cleaning, maintaining public health standards, conserving environmental conditions and sustainable financing. A legal structure in line with the domestic plan must also be established. Legislation is commonly formulated only as a manner of attributing liability. The legal structure should guarantee that the goals determined in the strategic documents are achieved in specific time frames. The structure must also make easier the design and implementation of the method; for example, the simplification of the idiom ‘solid waste’ should not be used to define responsibilities.

In addition, the framework should also supply information that can be helpful in addressing technical problems related to the selection of some waste administration systems. The legal structure must involve provisions that permit efficient application of the regulations.

Policy estimation includes the production of qualitative and quantitative evaluations of formal policy costs, through strategies to review the consequences of implementation and determine whether to update or discontinue them [41,42].

The latest insurance valuation literature, coupled with software programs from exceptional insurance valuation models, suggests real international interest in recognizing the practical outcomes and societal impact of waste control policies in anomalous countries [42]. Moreover, partnerships with major food service corporations or restaurants should be considered to develop innovative initiatives that go beyond simple acquiescence with the purpose. It should be determined whether the body responsible for insurance is the local authority, business, faculty, or nearby city and residents. In addition, it should decide whether the scope covers the environmental, financial, or social sectors. Also, it needs to determine whether the guarantee consists of policies for family waste types, recycling activities, transportation, waste reduction, infrastructure construction, waste types, use of beneficial waste, remedial standards, remedial methods, decomposition standards, etc., or pioneering standards. Moreover, methods in which households can be encouraged to recycle their wasted food must also be considered, and surveys must be conducted of behavior in nations where governments supply free bins for waste gathering.

### 4.5. Appropriate Technology

Variable objects, cloud services, applications and databases have been able to connect with the current system of other IoT devices, due to the growing interest in IoT. The IoT will result in a large number of new interfaces between new and existing systems, to provide solutions. The resulting information network will reduce costs and hazards, and improve business operations.

**Figure 10:**
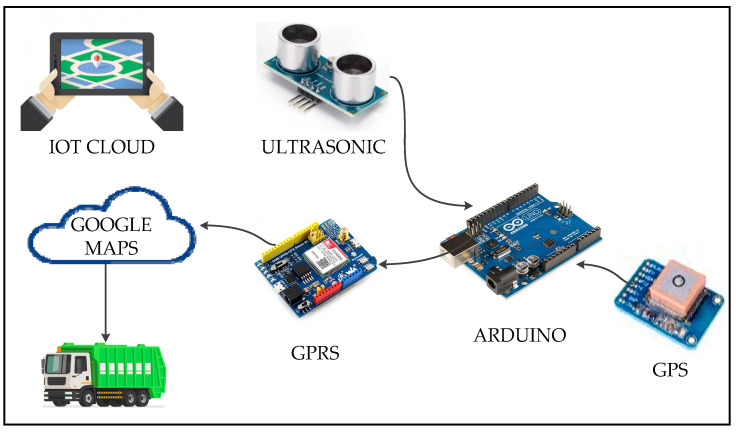
Integrated technology devices/applications.

IoT architecture has been proven to reduce operational costs of waste collection and to allow automation and the simplification of waste identification to enable recycling and waste treatment. Figure 10 shows an example of small devices/gadgets with a web-based system that allows the integration of the IoT as a new generation technology.

## 5. Experimental Results

These experiments were conducted for evaluating the proposed transportation algorithm. The experiment was performed using C++ by creating a random transportation map with a specified number of smart bins and relevant information, including bin status. The status values were randomly assigned for every smart bin, to maintain conformance. The most efficient routes for different transportation road maps were chosen on the basis of the GA. Different routes were evaluated to find the most efficient route from the truck source station to the selected smart bins, which are the smart bins that most urgently need to be cleaned. In this work, the evaluation experiments were executed for one collect truck station with id: 0 and all smart bins on the map as destinations. This experiment was performed with two constraints: *R_fs_* (residual free space) and *V_ip_* (importance priority value). In this simulation, *R_fs_* was fixed as the maximum (50%), and *V_ip_* was fixed as the maximum (50). 

Priority was given based on the waste source locations, such as homes, hospitals, retail, restaurants, and supermarkets, in addition to the danger of the waste and the significance of the site. Table 1 shows the available routes to selected smart bin nodes, where the residual free space for each route varies.

### Path Selection with Priority

In this experiment, organic waste that was collected along a predetermined route was avoided. A predetermined route leads to unnecessary costs and a waste of equipment. Full containers are often collected first. The priority of the waste type is the constraint that reduces leakage of waste containers into the street or in large quantities. This spilled waste can lead to increased cleaning costs, health risks, and resident complaints.

**Figure 11:**
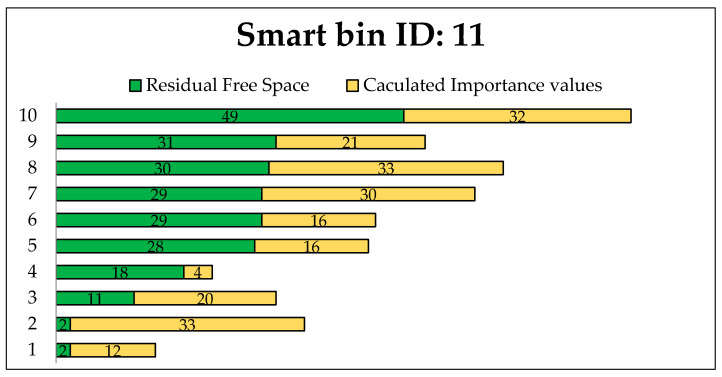
Evaluation routes to smart bin id: 11.

**Figure 12:**
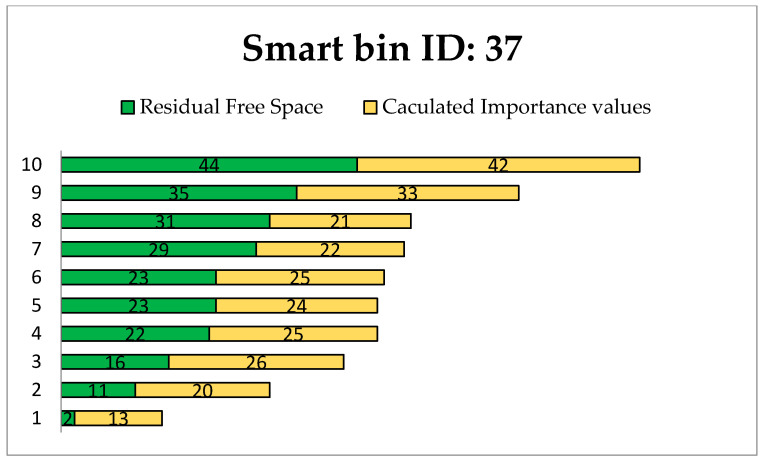
Evaluation routes to smart bin id: 37.

**Figure 13:**
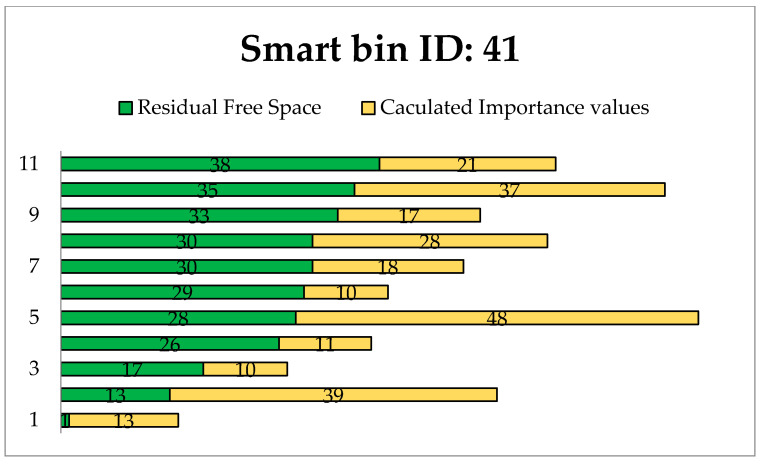
Evaluation routes to smart bin id: 41.

Figure 11, Figure 12 and Figure 13 present the calculated constraints for all routes to the smart bins with 11, 37 and 41, respectively, among the 50 smart bins randomly distributed in this experiment.

We can see different available routes to each destination smart bin. The residual free space is the main factor when selecting an efficient route. The most efficient route is determined based on the minimum free space in addition to the importance priority value.

Table 2 shows the result of the simulation of finding an efficient route from the truck station to other selected smart bins with equal priority of residual free space and importance for cleaning the bins, randomly distributed on the transportation map.

**Figure 14:**
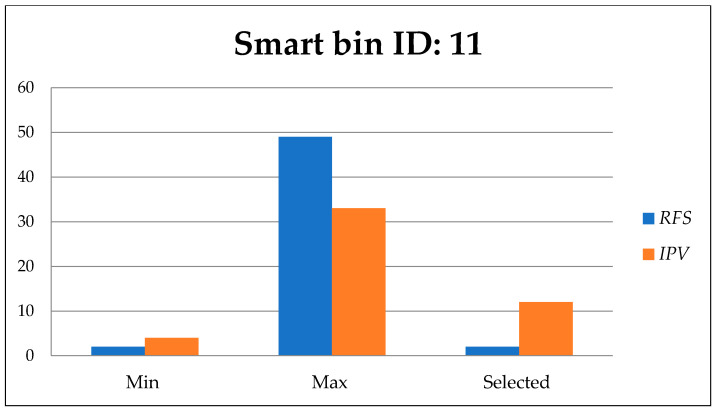
Residual free space *R_fs_*, and importance priority value *IPv* to smart bin id: 11.

**Figure 15:**
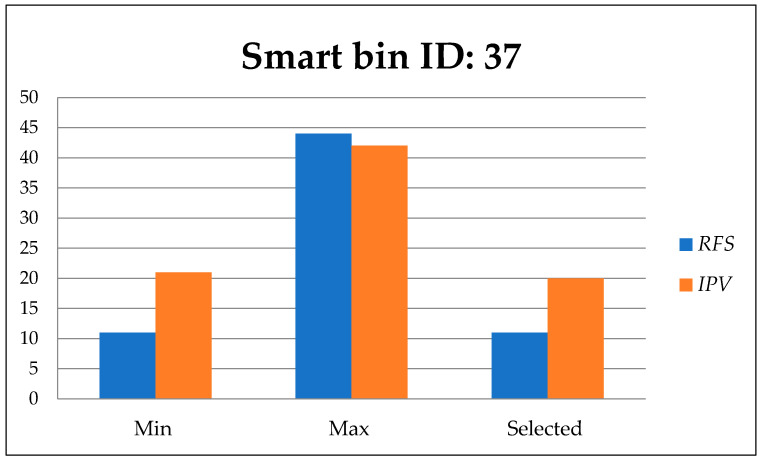
Residual free space *R_fs_*, and importance priority value *IPv* to smart bin id: 37.

**Figure 16:**
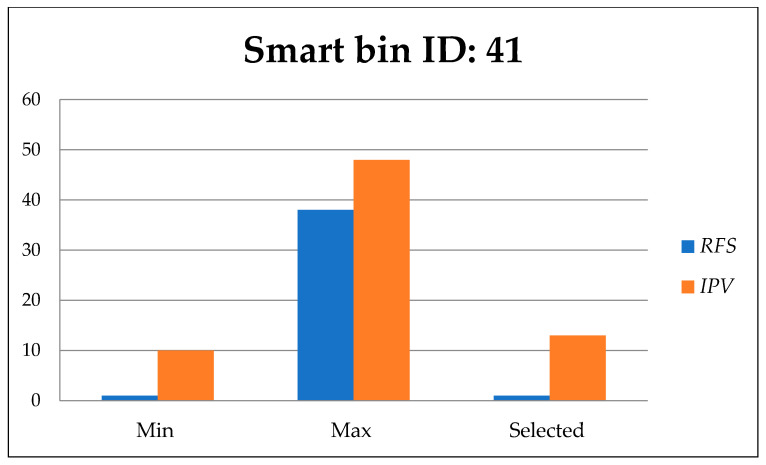
Residual free space *R_fs_*, and importance priority value *IPv* to smart bin id: 41.

Figure 14, Figure 15 and Figure 16 show the minimum and maximum cumulative residual free space *R_fs_,* and importance priority values *IPv* for each route directed to the urgent smart bins that send a call to clean the bin. The selected values for *R_fs_* and *IPv* are the efficient values.

Figure 14 presents the minimum and maximum values of residual free space *R_fs_*, and importance priority value *IPv* among 10 available routes including the selected route to smart bin 11.

In Figure 15, the minimum and maximum values of residual free space *R_fs_*, and importance priority value *IPv* are presented over 9 available routes including the selected route to smart bin 37.

Figure 16 presents the minimum and maximum values of residual free space *R_fs_*, and importance priority value *IPv* among 11 available routes including the selected route to smart bin 41.

The most efficient route was selected based on the *R_fs_* and *IPv* to find the most efficient path to reach the target smart bin. Based on the same priority, Table 3 presents the efficient of the selected *R_fs_* and *IPv* values. The selected route to the target smart bin that sends a clean bin request includes each individual smart bin that needs to be cleaned. It is clear that the selected route includes the smart bins that most urgently need to be cleaned on the way to the target smart bin. Thus, the advantage of using the same priority for *R_fs_* and *IPv* is shown in the selected route that has efficient values compared to the other available routes.

In all three selected smart bins, when a call for waste truck collection is considered, the proposed algorithm assumes that all constraints have the same priority in the absence of a priority case. Thus, the experiment demonstrates that the proposed algorithm is sufficient to find the most efficient route and collect the most urgent smart bins required for waste collection, by using GA based on the residual free space and importance priority.

## 6. Conclusions

‘Sustainability’ is a buzzword these days. This word is used in many contexts by everyone from goods manufacturers and service providers to international politicians. However, it remains unclear whether all these stakeholders understand the term ‘sustainable development’ in the same sense. The current rate of resource extraction is predicted to be 10,000 times higher than the rate of natural resource production. This ratio may significantly change in the near future. Moreover, it is not clear whether the WMS system can make a meaningful contribution. Accordingly, a more sustainable OWM than a WMS must be developed for a sustainable society. Organic waste accounts for more than 50% of global MSW. The EAP region produces the most organic waste, accounting for approximately 62%, followed by the Middle East and North Africa (61%). Global waste reduction is a main goal of scientists through various research methods. Several recommendations have been made to control organic waste, including composting and making biochar and bioenergy for cosmetic, pharmaceutical, and food supplements. The proposed integrated system is compatible with government, public organizations, and the environment. The country of Saudi Arabia is a complex cultural mix, a combination that is truly reflected in the OWM system. Countries like Japan demonstrate a sustainable approach to MSW management. Meanwhile, countries like China are struggling to meet new needs due to aggressive development. In every country, organic waste management is an important, ongoing, and meaningful public service system that must be effectively provided to the community to maintain aesthetic and public health standards. Municipal agencies need to plan and manage these systems during increasing urbanization and population growth. 

Today, KSA can demonstrate a sustainable OWM system through the proposed integrated approach. Systematic efforts are needed to improve various factors, including governance by using law and policy, education by institutional arrangements, appropriate technologies, and public community participation in system proposals. Various waste control models have been produced for the use of particular IoT devices. Various models use IoT devices, which include capacity, weight, temperature, cup cheer, and chemical sensors, relying on the popularity of those devices. A genetic-based waste collection transportation algorithm is proposed, using IoT technology and a computational algorithm to speed up waste collection and select the most efficient transportation path that improves the efficiency of waste collection truck management. The paths selected based on the residual free space *R_fs_*, and importance priority value *IPv* were the most efficient routes among those available to each selected smart bin destination. The *R_fs_* of selected routes were less than the maximum *R_fs_* of available routes by 4.08%, 25%, and 2.63% for smart bins 11, 37, and 41 respectively. The *IPv* of selected routes was less than the maximum *IPv* of available routes by 36.36%, 47.61%, and 27.08% for smart bins 11, 37, and 41 respectively. However, the proposed integrated approach, including the waste collection transportation algorithm, would be helpful over a variety of country-specific layouts. In future work, smart containers will be implemented and integrated with the proposed garbage transport algorithm. It should consider more criteria for path selection, such as distance, safety, accident-free status, and any other useful criteria.

## Figures and Tables

**Table 1:** Experiment scenarios.

Call from Smart Bin ID	List of Smart Bins in the Route	Residual Free Space %	Importance Priority
11	0 15 11	2	12
	0 31 11	2	33
	0 23 11	11	20
	0 26 11	18	4
	0 27 11	28	16
	0 21 47 22 11	29	16
	0 36 40 11	29	30
	0 6 10 11	30	33
	0 27 16 11	31	21
	0 23 38 18 16 26 11	49	32
37	0 15 37	11	20
	0 14 37	16	26
	0 23 37	22	25
	0 45 37	23	24
	0 19 37	23	25
	0 36 9 37	29	22
	0 35 37	31	21
	0 38 45 37	35	33
	0 48 34 3 37	44	42
41	0 15 41	1	13
	0 31 11 41	13	39
	0 14 41	17	10
	0 27 41	26	11
	0 6 24 41	28	48
	0 26 11 41	29	10
	0 11 41	30	18
	0 26 5 41	30	28
	0 6 41	33	17
	0 15 36 48 41	35	37
	0 32 14 41	38	21

**Table 2:** Efficient route selected.

Smart Bin Target	List of Smart Bins in the Selected Route (*R_fs_* Priority)	List of Smart Bins in the Selected Route (Equal Priority)
11	0 15 11	0 15 11
37	0 15 37	0 14 37
41	0 15 41	0 15 41

**Table 3:** Residual free space *R_fs_*, and importance priority value *IPv*.

Call from Smart Bin ID	Residual Free Space %			Importance Priority		
	Min	Max	Selected	Min	Max	Selected
11	2	49	2	4	33	12
37	11	44	11	21	42	20
41	1	38	1	10	48	13

## Data Availability

Data is contained within the article.
